# Parthanatos participates in glutamate‐mediated HT22 cell injury and hippocampal neuronal death in kainic acid‐induced status epilepticus rats

**DOI:** 10.1111/cns.13934

**Published:** 2022-07-31

**Authors:** Xue Wang, Wuqiong Zhang, Pengfei Ge, Miaomiao Yu, Hongmei Meng

**Affiliations:** ^1^ Department of Neurology and Neuroscience Center First Hospital of Jilin University Changchun Jilin People's Republic of China; ^2^ Department of Neurology Beijing Friendship Hospital, Capital Medical University Beijing People's Republic of China; ^3^ Department of Neurology First Hospital of Jilin University Changchun Jilin People's Republic of China

**Keywords:** epilepsy, glutamate, PARP‐1, parthanatos, ROS

## Abstract

**Aims:**

Epileptic seizures or status epilepticus (SE) can cause hippocampal neuronal death, which has detrimental effects. Parthanatos, a new form of programmed cell death, is characterized by hyperactivation of poly (ADP‐ribose) polymerase‐1 (PARP‐1), excessive synthesis of poly ADP‐ribose polymer, mitochondrial depolarization, and nuclear translocation of apoptosis‐inducing factor, observed in various neurodegenerative disorders but rarely reported in epilepsy. We aimed to investigate whether parthanatos participates in the mechanism of seizure‐induced hippocampal neuronal death.

**Methods:**

Glutamate‐mediated excitotoxicity cell model was used to study the mechanism of seizure‐induced cell injury. Injection of kainic acid into the amygdala was used to establish the epileptic rat model. Corresponding biochemical tests were carried out on hippocampal tissues and HT22 cells following indicated treatments.

**Results:**

In vitro, glutamate time‐dependently induced HT22 cell death, accompanied by parthanatos‐related biochemical events. Pretreatment with PJ34 (PARP‐1 inhibitor) or small interfering RNA‐mediated PARP‐1 knockdown effectively protected HT22 cells against glutamate‐induced toxic effects and attenuated parthanatos‐related biochemical events. Application of the antioxidant N‐acetylcysteine (NAC) rescued HT22 cell death and reversed parthanatos‐related biochemical events. In vivo, PJ34 and NAC afforded protection against SE‐induced hippocampal neuronal damage and inhibited parthanatos‐related biochemical events.

**Conclusion:**

Parthanatos participates in glutamate‐induced HT22 cell injury and hippocampal neuronal damage in rats following epileptic seizures. ROS might be the initiating factor during parthanatos.

## Graphical abstract

The present study demonstrated that parthanatos participated in glutamate‐induced HT22 cell injury and hippocampal neuronal damage in rats following epileptic seizures. In addition, ROS might be the initiating factor in the process of parthanatos. The findings highlight the robust neuroprotective potential of pharmacological interventions targeting PARP activity in epilepsy and provide multiple potential therapeutic targets that need to be verified in future studies.

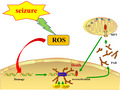

## 1 INTRODUCTION

Epilepsy is a common chronic neurological disease that affects more than 68 million individuals worldwide.^1^ Prolonged epileptic seizures or status epilepticus (SE) can induce different degrees of brain damage, particularly in the hippocampus. Hippocampal neurons are sensitive to epileptic discharge and prone to death. Seizure‐induced neuronal death promotes the development of epilepsy and leads to hippocampal sclerosis (HS).^2^ In addition, hippocampal neuronal damage is closely related to cognitive impairment following epileptic seizures.^3^ Therefore, it is crucial to elucidate molecular mechanisms underlying seizure‐induced neuronal death, which could potentially help ameliorate the serious impact of seizures on the brain and limit further development of epilepsy.

Poly (ADP‐ribose) polymerase‐1 (PARP‐1)‐dependent cell death, also called parthanatos, is a special cell death characterized by hyperactivation of PARP‐1, excessive synthesis of the poly ADP‐ribose (PAR) polymer, mitochondrial depolarization, and nuclear translocation of apoptosis‐inducing factor (AIF).^4^ PARP‐1 is a ribozyme that maintains the stability of the intracellular environment and facilitates DNA damage repair, transcriptional regulation, inflammation, metabolism, oncogene‐related signal transduction, and cell differentiation and death.^5^ PARP‐1 is directly activated by DNA damage caused by reactive oxygen species (ROS), alkylating agents, light, and ultraviolet (UV) rays.^6^, ^7^, ^8^ Overactivation of PARP‐1 can rapidly deplete NAD+ and ATP, promoting excessive synthesis and accumulation of PAR polymer, which further induces AIF translocation from the mitochondria into the nucleus, resulting in bioenergetic collapse and cell death via inhibition of glycolytic enzyme hexokinase.^9^, ^10^ Accumulating evidence supports the involvement of parthanatos in various neurological disorders. Inhibition of PARP‐1 was found to afford neuroprotection in stroke, excitotoxic stress, Parkinson's disease, Alzheimer's disease, and traumatic brain injury.^11^, ^12^, ^13^, ^14^, ^15^, ^16^ However, the role of parthanatos in the mechanism of seizure‐induced neuronal death remains unknown.

Glutamate is a primary mediator of the excessive excitatory neurotransmission induced by neuronal excitotoxicity.^17^ High concentrations of hippocampal extracellular glutamate activate the N‐methyl‐D‐aspartate receptor, facilitating Ca^2+^ entry into neuronal cells.^18^ Elevated intracellular Ca^2+^ elicits ROS production, and the subsequent ROS accumulation can oxidize DNA, lipids, and proteins, thereby inducing cellular damage.^19^ Given that DNA damage directly mediates PARP‐1 activation, we speculate that parthanatos might be involved in the mechanism of seizure‐induced neuronal death and that ROS might be an important initiating factor in the process of parthanatos. Accordingly, we examined the role of PARP‐1 in glutamate‐mediated HT22 cell injury in vitro and SE‐induced hippocampal neuronal damage in vivo and explored the potential underlying mechanism.

## 2 MATERIALS AND METHODS

### 2.1 Cell lines and culture

HT22 cells were thawed and then cultured in Dulbecco's modified Eagle's medium containing 10% fetal bovine serum and 1% penicillin–streptomycin. The cells were incubated in 5% CO_2_ at 37°C and used in the mid‐log phase for subsequent experiments.

### 2.2 CCK8 assay

HT22 cells (5 × 10^3^ cells/well) were seeded in 96‐well microplates and incubated for 24 h. To induce a glutamate‐mediated excitotoxicity cell model, cellular viability was examined at different glutamate doses (0, 5, 10, and 20 mM) for different incubation periods (6, 12, and 24 h) using the CCK8 assay (Dojindo) according to the manufacturer's instructions.

### 2.3 Cell treatment and transfection of small interfering RNA (siRNA)

Briefly, HT22 cells were incubated with 10 mM glutamate for different periods (6, 12, and 24 h) to induce cell injury. To induce parthanatos inhibition, HT22 cells were treated with 20 μmol/L PARP‐1 inhibitor PJ34 (N‐[6‐oxo‐5,6‐dihydro‐phenanthridin‐2‐yl]‐N, N‐dimethylacetamide HCl) (Selleck) for 1 h, followed by glutamate incubation. To achieve ROS inhibition, cells were treated with antioxidant N‐acetylcysteine (NAC; 3 mmol/L; Sigma‐Aldrich) for 1 h, followed by glutamate incubation.

HT22 cells (5 × 10^3^ cells/well) were seeded onto a 10‐cm culture dish. Lipofectamine 2000 (Invitrogen) was used to transfect siRNA according to the manufacturer's instructions. Following overnight transfection of cells with siRNA, glutamate was added to cells at indicated doses for subsequent experiments.

### 2.4 Lactate dehydrogenase (LDH) cytotoxicity assay

Briefly, HT22 cells (5 × 10^3^ cells/well) were seeded in 96‐well microplates and incubated for 24 h. After treatment with corresponding compounds at indicated concentrations, an LDH cytotoxicity assay kit (Beyotime Biotech) was used to evaluate cytotoxicity at 490 nm, according to the manufacturer's protocol.

### 2.5 Immunofluorescence staining

Briefly, HT22 cells were treated with glutamate for 24 h and then fixed in 4% paraformaldehyde. Cells were washed with phosphate‐buffered saline (PBS) and then incubated with 1% Triton X‐100 for 10 min. After blocking with 5% bovine serum albumin (BSA) for 10 min, cells were incubated with primary antibodies against PAR (1:100; Millipore) and AIF (1:100; Abcam) overnight at 4°C. Subsequently, cells were incubated with fluorescence‐conjugated secondary antibodies against mouse (1:200; Abcam) or rabbit (1:200; Abcam) for 1 h at room temperature. After counterstaining the nuclei with DAPI (Beyotime Biotech) for 5 min, the cells were visualized under a fluorescence microscope (Olympus IX71).

### 2.6 Mitochondrial membrane potential (JC‐1) assay

Briefly, HT22 cells treated with glutamate at indicated doses for different periods and pretreated for 1 h with 20 μmol/L PJ34 or subjected to siRNA‐mediated PARP‐1 knockdown overnight were stained with JC‐1 (Beyotime Biotech), as described by Ma et al.^20^ Cells were then analyzed using flow cytometry (CytoFLEX S, Beckman Coulter Inc.).

### 2.7 Measurement of ROS production

The level of intracellular ROS in HT22 cells incubated with glutamate alone or treated with corresponding compounds was evaluated according to the instructions of the redox‐sensitive dye DCFH‐DA (Beyotime Biotech). Cells were washed twice with PBS, stained with DCFH‐DA (20 μmol/L) for 30 min in the dark, and then analyzed by flow cytometry (CytoFLEX S, Beckman Coulter Inc.). Cells were visualized under a fluorescence microscope (Olympus IX71).

### 2.8 Measurement of 8‐hydroxy‐2 deoxyguanosine (8‐OHdG) level

8‐OHdG is a common biomarker of DNA damage and reflects the level of cellular DNA oxidative damage. 8‐OHdG in cells was examined following the instructions of the 8‐OHdG detection ELISA kit (Nanjing Jiancheng Bioengineering Institute).

### 2.9 Measurement of glutathione (GSH) level

HT22 cells in different groups were collected by centrifugation (800 g, 5 min), twice washed with PBS, and then subjected to centrifugation (10,000 *g*, 10 min, 4°C) to obtain the supernatant for detecting the intracellular total GSH level. The level of intracellular total GSH was detected by enzyme labeling following the DTNB‐GSSH reductase recycling assay kit (Beyotime Biotechnology) instructions at 405 nm.

### 2.10 Animals

Male Wistar rats (280–300 g) were obtained from the animal center of the Jilin University. All experimental rats had access to water and normal rat chow ad libitum under standard conditions (12‐h light/dark cycle, 23–25°C). Experimental protocols were approved by the Institutional Animal Care and Ethics Committee of Jilin University, China.

### 2.11 Epileptic models and drug administration

Epileptic models were induced by administering kainic acid (KA; Abcam, UK) according to a previously established standard method.^21^ In total, 72 rats were randomly divided into 4 groups:

Sham group (*n* = 18): PBS was injected into the amygdala of rats.

KA+ vehicle group (*n* = 18): Epileptic rats were treated with the vehicle (PBS).

KA+ PJ34 group (*n* = 18): Epileptic rats were treated with PJ34 (15 mg/kg/day, 3 days before and 3 days after seizure induction).

KA+ NAC group (*n* = 18): Epileptic rats were treated with NAC (200 mg/kg/day, 3 days before and 3 days after seizure induction).

### 2.12 Pathological assessments

On Day 3 post‐SE induction, rats from different groups (*n* = 6 per group) were anesthetized and decapitated, and brain tissues were extracted to prepare frozen sections at a thickness of 30 μm. Fluoro‐Jade B (FJB) staining (Millipore) was used to detect degenerating neurons, as previously described.^21^ A fluorescence microscope (Olympus IX71) was used to visualize the number of FJB‐positive cells.

### 2.13 Western blot analysis

Hippocampal tissues and HT22 cells were collected according to standard protocols. Total protein was extracted using RIPA lysis buffer (Beyotime Biotech) containing 1% proteinase inhibitors (Beyotime Biotech). Nuclear and cytoplasmic proteins were isolated using a Nuclear and Cytoplasmic Protein Extraction Kit (Beyotime Biotech). Mitochondrial proteins from HT22 cells and hippocampal tissues were isolated following the instructions of the Cell Mitochondria Isolation Kit (Beyotime Biotech) and Tissue Mitochondria Isolation Kit (Beyotime Biotech), respectively.

A BCA Protein Assay Kit (Beyotime Biotech) was used to quantify the protein concentration. Protein samples were electrophoresed on 10% sodium dodecyl sulfate gels and then transferred to polyvinylidene difluoride membranes (Millipore). After blocking with 5% BSA for 1 h, the membranes were incubated with anti‐PARP‐1 (1:1000, Abcam), anti‐PAR (1:200, Millipore), anti‐AIF (1:1000, Abcam), anti‐β‐actin (1:1000, Abcam), anti‐COX IV (1:1000, Abcam), and anti‐Histone H3 (1:1000, Abcam) at 4°C overnight. After incubation with horseradish (HRP)‐conjugated secondary antibodies against mouse (1:3000; Abcam) or rabbit (1:3000; Abcam) for 1 h, the membranes were washed with PBS and analyzed using the Odyssey infrared imaging system (Li‐COR). Quantity One software was used to quantify protein bands.

### 2.14 Statistical analysis

All experimental data are expressed as mean ± standard deviation (SD) and were analyzed using SPSS (version 22.0; IBM) and GraphPad Prism 8.0 (GraphPad Software, Inc.). Data were tested for normality using the Kolmogorov–Smirnov (K‐S) test. Student's t test or one‐way ANOVA was used for comparisons of the normal data, and the Mann–Whitney U test was used for comparisons of the non‐normal data. *p* values were corrected using Bonferroni's test. Statistical significance was set as follows: *p < 0.05 and **p < 0.01.

## 3 RESULTS

### 3.1 Effects of glutamate on HT22 cells

**FIGURE 1:**
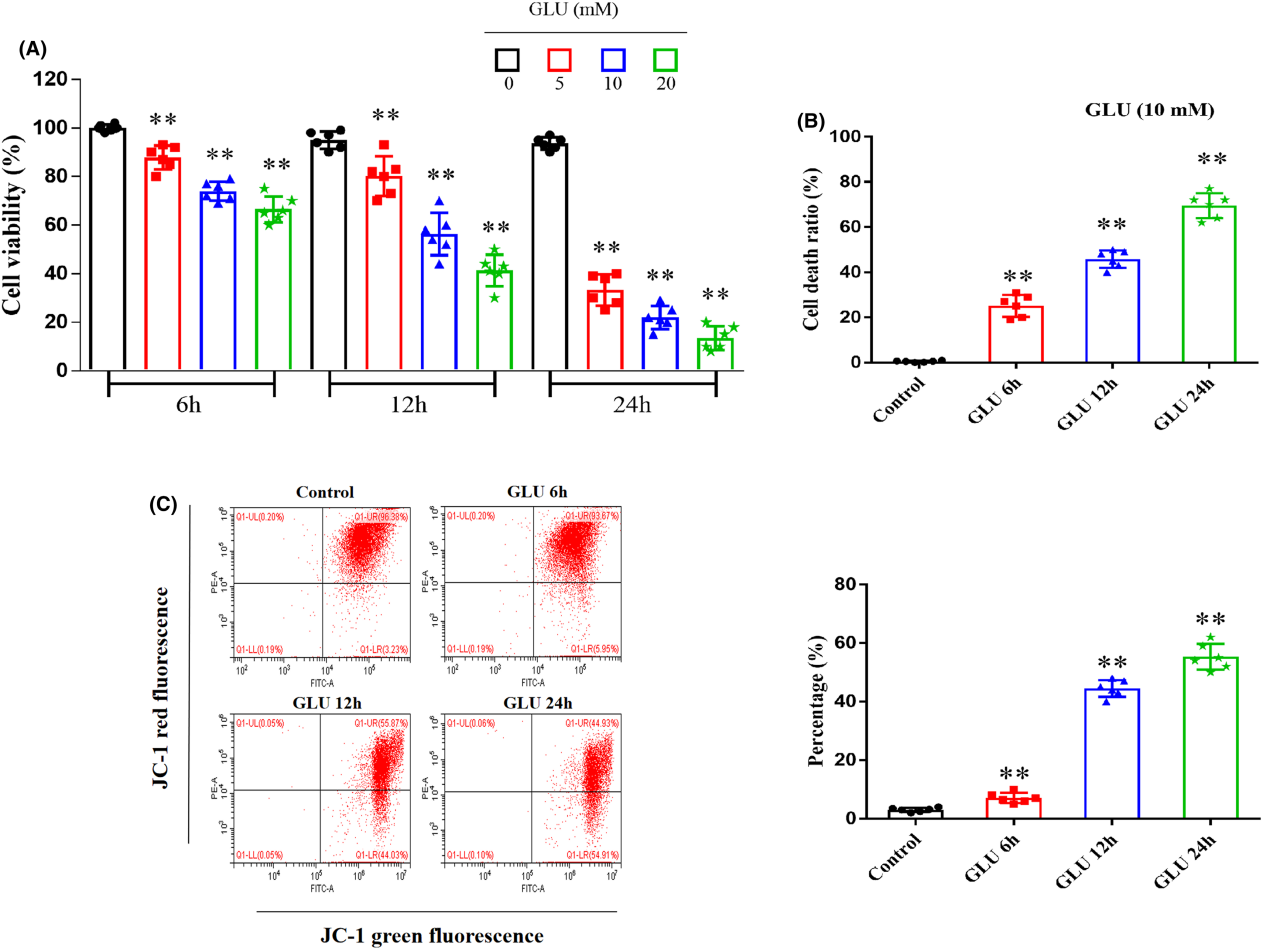
Effects of glutamate on HT22 cells. (A) Glutamate decreases HT22 cell viability in a dose‐ and time‐dependent manner. (B) The cell death ratio of HT22 cells induced by 10 mM glutamate progressively increases following incubation for 6, 12, and 24 h. (C) The glutamate‐induced declining percentage of mitochondrial membrane potentials in HT22 cells progressively increases following the incubation for 6, 12, and 24 h. Data are expressed as the mean ± standard deviation (SD; **p < 0.01).

The CCK8 assay was performed to detect the viability of HT22 cells incubated with various glutamate concentrations for different periods and examine glutamate‐mediated toxic effects on HT22 cells. As shown in Figure 1A, the average viability of HT22 cells decreased significantly with increasing glutamate concentrations and prolonged incubation periods. Treatment with 10 mM glutamate for 12 h induced approximately 50% cell death (*p* < 0.01; Figure 1A).

Using lactate dehydrogenase cytotoxicity assay, glutamate (10 mM) was selected to determine the cell death ratio following different incubation periods. The cell death ratio increased to 25.2 ± 4.8 (*p* < 0.01), 45.8 ± 3.9 (*p* < 0.01), and 69.5 ± 5.6% (*p* < 0.01) after HT22 cell incubation with glutamate for 6, 12, and 24 h, respectively (Figure 1B).

Furthermore, the effects of glutamate on mitochondrial membrane potential in HT22 cells were detected by JC‐1 staining. Based on flow cytometric analysis, the percentage of mitochondrial membrane potentials in HT22 cells reduced to 7.08 ± 1.74 (*p* < 0.01), 44.50 ± 2.88 (*p* < 0.01), and 55.33 ± 4.46% (*p* < 0.01) after incubation with glutamate for 6, 12, and 24 h when compared to that in the control group (3.03 ± 0.71%; Figure 1C).

### 3.2 Glutamate altered parthanatos‐related protein expression in HT22 cells

**FIGURE 2:**
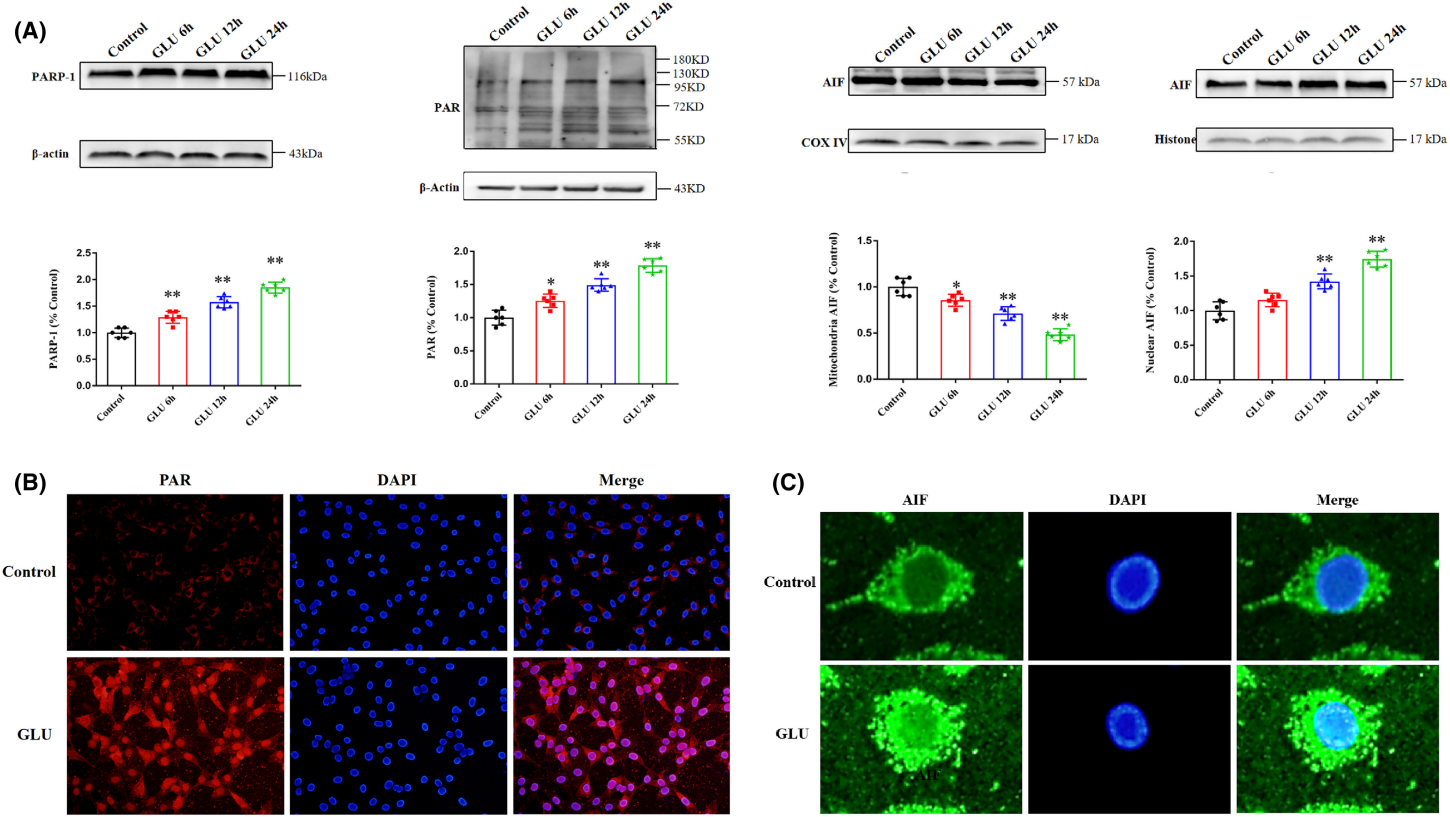
Glutamate alters parthanatos‐related protein expression. (A) Western blot analysis of PARP‐1, PAR polymer, and mitochondrial and nuclear AIF levels in HT22 cells following incubation with glutamate for different periods (6, 12, and 24 h). β‐actin, COX IV, and histone were used as cytoplasmic, mitochondrial, and nuclear loading controls, respectively. (B) Immunofluorescent analysis of PAR polymer accumulation (red) in HT22 cells incubated with glutamate. (C) Immunofluorescent analysis of AIF (green) translocation to the nucleus in HT22 cells incubated with glutamate. Nuclei were stained with DAPI (blue). Scale bars: 50 μm. Data are expressed as mean ± standard deviation (SD; *p < 0.05, **p < 0.01). AIF, apoptosis‐inducing factor; PARP‐1, poly (ADP‐ribose) polymerase‐1.

Western blotting was performed to detect parthanatos‐related protein expression and clarify whether glutamate initiated parthanatos expression in HT22 cells. As shown in Figure 2, the expression levels of PARP‐1 and PAR polymers were significantly elevated in a time‐dependent manner, and AIF translocation from the mitochondria to the nucleus progressively increased with prolonged incubation (6, 12, and 24 h) of HT22 cells with glutamate (Figure 2A). Excessive PAR polymer synthesis and AIF translocation to the nucleus in glutamate‐treated HT22 cells were visualized using immunofluorescence (Figure 2B,C). These results suggested that parthanatos may participate in glutamate‐induced HT22 cell damage.

### 3.3 Inhibition of PARP‐1 alleviated glutamate‐induced parthanatos in HT22 cells

**FIGURE 3:**
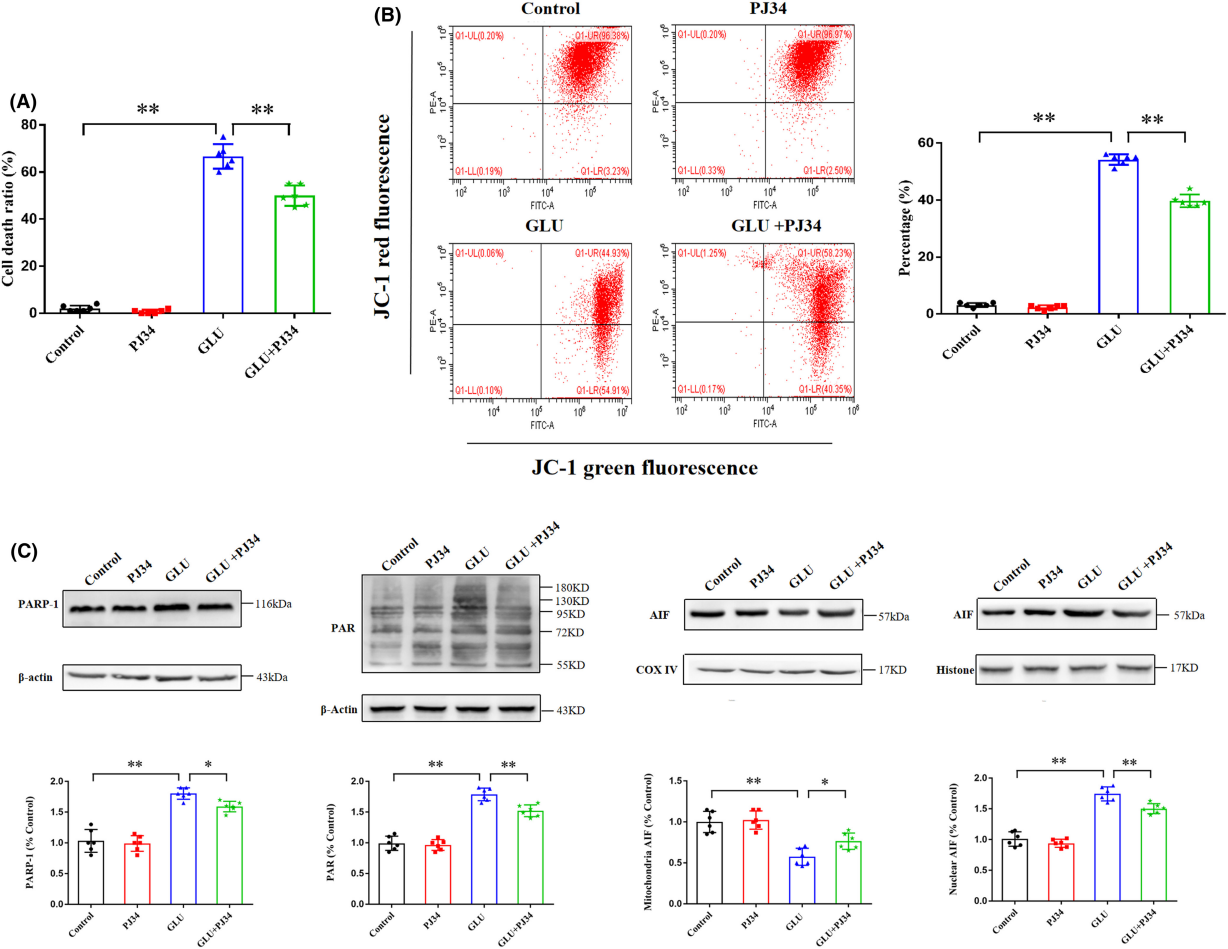
PARP‐1 inhibitor PJ34 ameliorates glutamate‐induced parthanatos in HT22 cells. (A) PJ34 ameliorates the death ratio of HT22 cells induced by glutamate. (B) PJ34 inhibits the declining percentage of mitochondrial membrane potentials in HT22 cells induced by glutamate. (C) Glutamate induces upregulation of PARP‐1, accumulation of PAR polymer, and AIF translocation from mitochondria to the nucleus in HT22 cells; PJ34 significantly inhibits these effects. Data are expressed as mean ± standard deviation (SD; *p < 0.05, **p < 0.01). AIF, apoptosis‐inducing factor; PARP‐1, poly (ADP‐ribose) polymerase‐1.

The PARP‐1 inhibitor PJ34 was administered to determine the role of PARP‐1 in glutamate‐induced HT22 cell death. As shown in Figure 3, pretreatment with PJ34 reduced the cell death ratio following glutamate‐induced damage in HT22 cells from 66.7 ± 5.2% to 50.0 ± 4.3% (*p* < 0.01; Figure 3A) and inhibited the declining percentage of mitochondrial membrane potential from 55.3 ± 3.6% to 39.7 ± 3.1% (*p* < 0.01; Figure 3B). At the protein level, pretreatment with PJ34 significantly inhibited glutamate‐induced increased PARP‐1 and PAR polymer expression and AIF translocation from the mitochondria to the nucleus in HT22 cells (Figure 3C). Therefore, these results indicated that PJ34‐mediated PARP‐1 inhibition could protect HT22 cells against the lethal effects of glutamate and attenuate changes in parthanatos‐related proteins.

### 3.4 PARP‐1 knockdown alleviated glutamate‐induced parthanatos in HT22 cells

**FIGURE 4:**
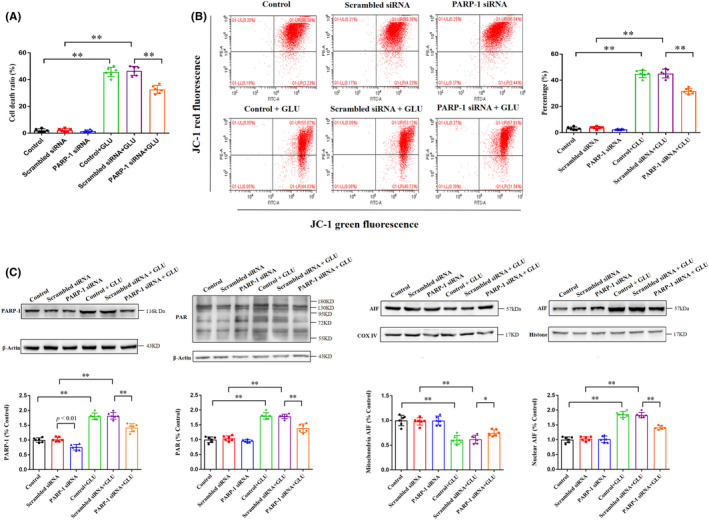
siRNA‐mediated knockdown of PARP‐1 attenuates glutamate‐induced HT22 cell injury. (A) siRNA‐mediated PARP‐1 knockdown rescues glutamate‐induced HT22 cell death. (B) siRNA‐mediated PARP‐1 knockdown inhibits the declining percentage of mitochondrial membrane potentials in HT22 cells induced by glutamate. (C) siRNA‐mediated PARP‐1 knockdown inhibits the upregulated levels of PARP‐1, PAR polymer accumulation, and AIF translocation from mitochondria to the nucleus in glutamate‐induced HT22 cells. Data are expressed as mean ± standard deviation (SD; *p < 0.05, **p < 0.01). AIF, apoptosis‐inducing factor; PARP‐1, poly (ADP‐ribose) polymerase‐1.

siRNA transfection was performed to knock down PARP‐1 and further investigate the role of PARP‐1 in glutamate‐induced HT22 cell death. As shown in Figure 4, siRNA‐induced PARP‐1 knockdown decreased the cell death ratio (32.7 ± 3.0% vs. 46.5 ± 3.4%, *p* < 0.01; Figure 4A) and inhibited the declining percentage of mitochondrial membrane potentials (31.7 ± 2.2% vs. 45.2 ± 3.3%, *p* < 0.01; Figure 4B) in glutamate‐induced HT22 cells. Furthermore, siRNA‐mediated PARP‐1 knockdown significantly suppressed glutamate‐induced increased expression of PARP‐1 and PAR polymer, as well as AIF translocation from mitochondria to nuclei in HT22 cells (Figure 4C). These results suggested that PARP‐1 plays an important role in glutamate‐induced HT22 cell death.

### 3.5 ROS contributed to glutamate‐induced parthanatos in HT22 cells

**FIGURE 5:**
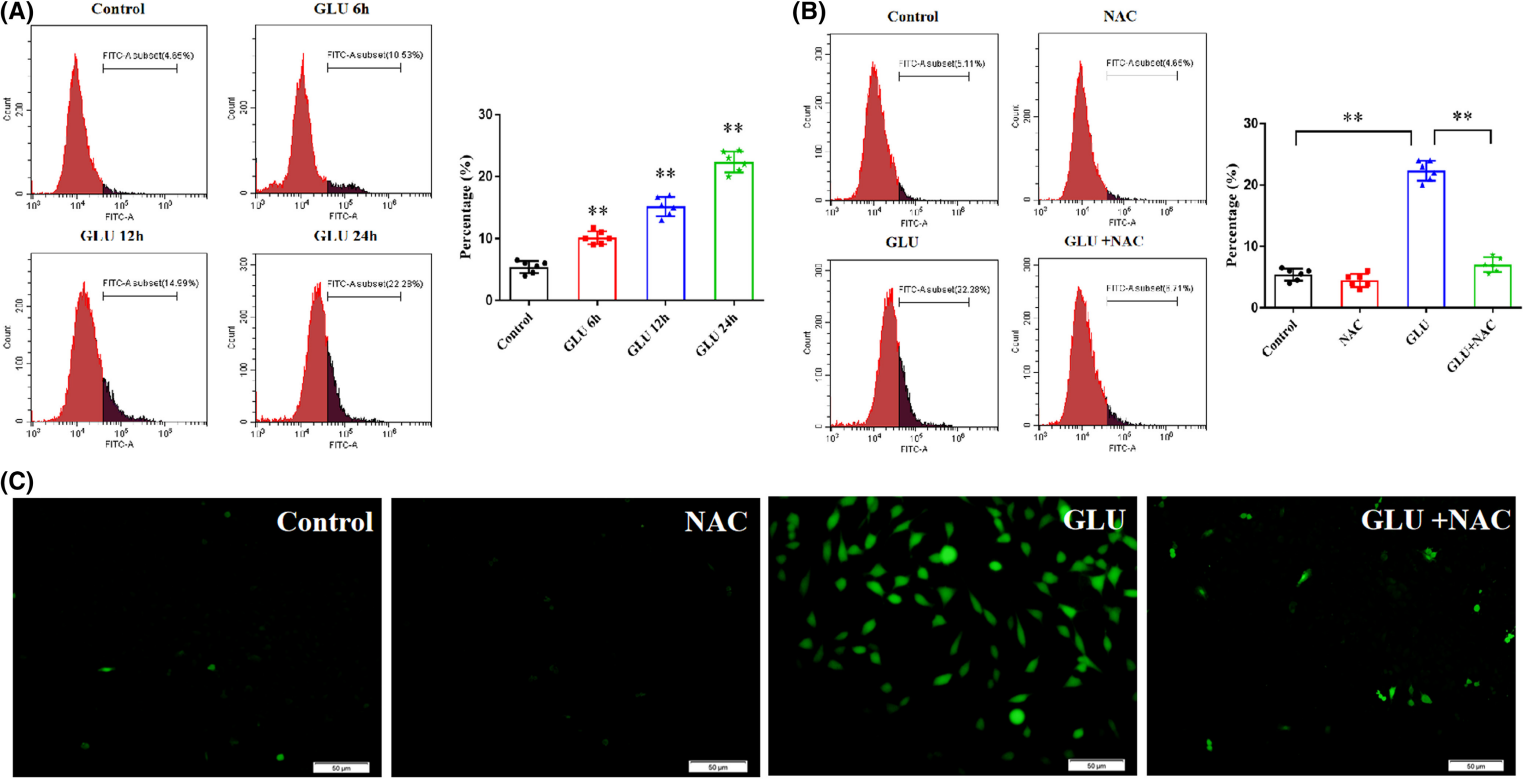
NAC decreases the level of ROS in glutamate‐induced HT22 cells. (A) ROS levels increase progressively in HT22 cells incubated with glutamate for 6, 12, and 24 h, as determined by flow cytometric analysis. (B) Pretreatment with antioxidant NAC attenuates ROS overproduction in glutamate‐induced HT22 cells. (C) As shown by DCFH‐DA staining, the green fluorescence in glutamate‐induced HT22 cells significantly increases, and NAC can decrease green fluorescence. Scale bars: 10 μm. Data are expressed as mean ± standard deviation (SD; **p < 0.01). NAC, N‐acetylcysteine; ROS, reactive oxygen species.

We next examined the role of ROS in glutamate‐induced HT22 cell death and the relationship between ROS and parthanatos. As shown in Figure 5A, compared with the control group, the ROS level increased to 10.15 ± 1.04 (*p* < 0.01), 15.18 ± 1.54 (*p* < 0.01), and 22.37 ± 1.68% (*p* < 0.01) after incubating HT22 cells with glutamate for 6, 12, and 24 h, respectively, as determined by flow cytometry. The antioxidant NAC attenuated ROS overproduction at 24 h in glutamate‐treated HT22 cells (*p* < 0.01; Figure 5B). DCFH‐DA staining showed that pretreatment with NAC decreased elevated levels of green fluorescence in glutamate‐treated HT22 cells (Figure 5C).

**FIGURE 6:**
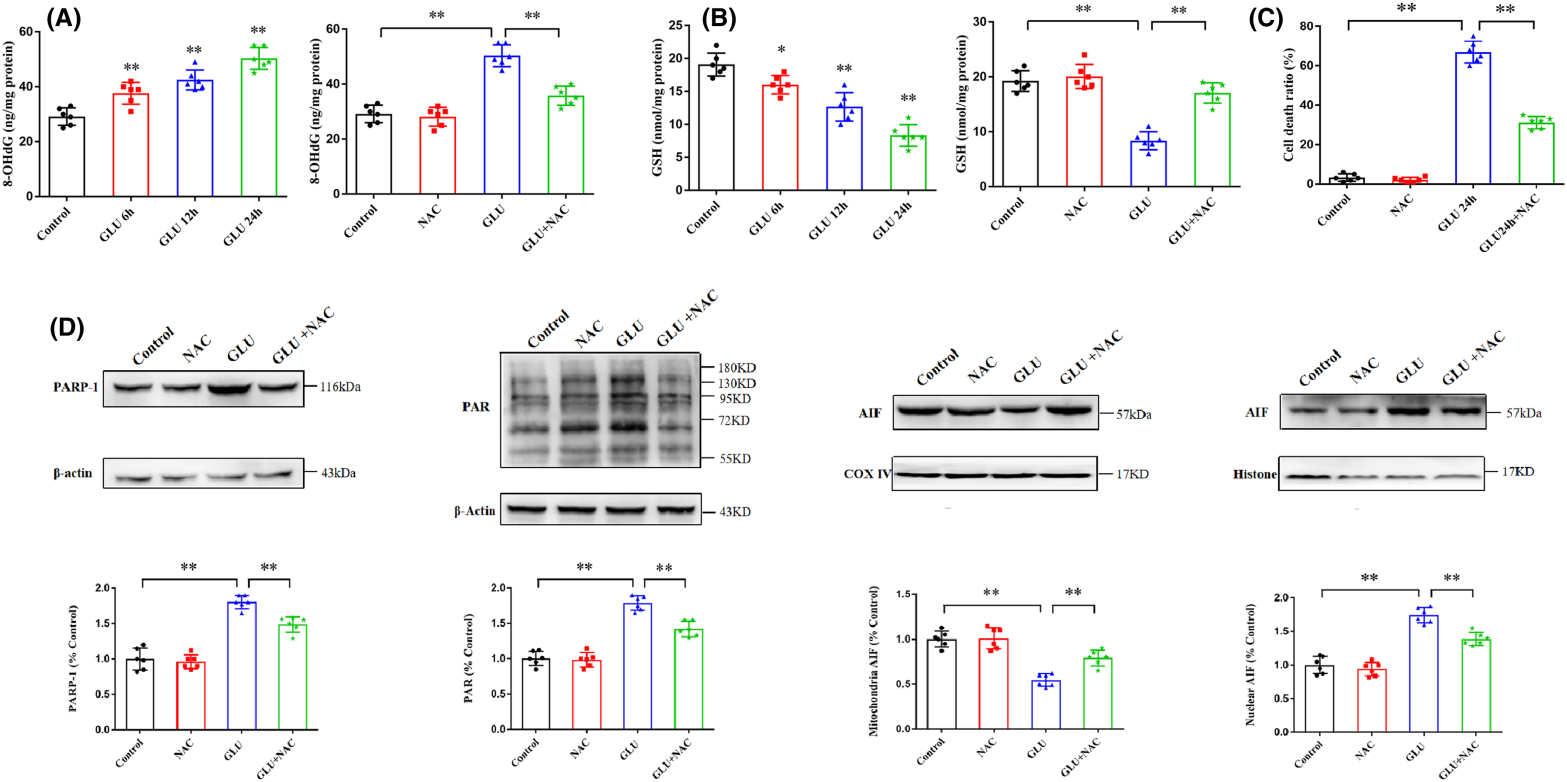
ROS contributes to glutamate‐induced parthanatos in HT22 cells. (A) The level of 8‐OHdG increases progressively following incubation of HT22 cells with glutamate for 6, 12, and 24 h. NAC effectively inhibits the increased level of 8‐OHdG in glutamate‐induced HT22 cells. (B) The level of GSH decreases progressively after incubating HT22 cells with glutamate for 6, 12, and 24 h. NAC effectively increases the level of GSH in glutamate‐induced HT22 cells. (C) Pretreatment with NAC decreases the cell death ratio of glutamate‐induced HT22 cells. (D) As determined by Western blot analysis, NAC significantly inhibits glutamate‐induced parthanatos‐related protein events. Data are expressed as mean ± standard deviation (SD; *p < 0.05, **p < 0.01). GSH, glutathione; NAC, N‐acetylcysteine.

Compared with the control group, the level of 8‐OHdG increased progressively in HT22 cells incubated with glutamate for 6 (*p* < 0.01), 12 (*p* < 0.01), and 24 h (*p* < 0.01; Figure 6A). Pretreatment with NAC effectively ameliorated the increased levels of 8‐OHdG following glutamate‐induced HT22 cell damage (*p* < 0.01; Figure 6A). Meanwhile, the GSH level steadily decreased in HT22 cells incubated with glutamate for 6 (*p* < 0.05), 12 (*p* < 0.01), and 24 h (*p* < 0.01) when compared to that in the control group (Figure 6B). Pretreatment with NAC effectively increased the GSH level in glutamate‐induced HT22 cells (*p* < 0.01; Figure 6B).

Incubation with glutamate induced HT22 cell death and increased the expression of PARP‐1 and PAR polymer and AIF translocation from the mitochondria to the nucleus in HT22 cells; NAC significantly inhibited these changes **(**Figure 6C,D). Therefore, these findings indicated that ROS could be an initial factor for PARP‐1 activation in glutamate‐induced HT22 cell death.

### 3.6 Treatment of PJ34 and NAC attenuated neuronal loss in epileptic rats

**FIGURE 7:**
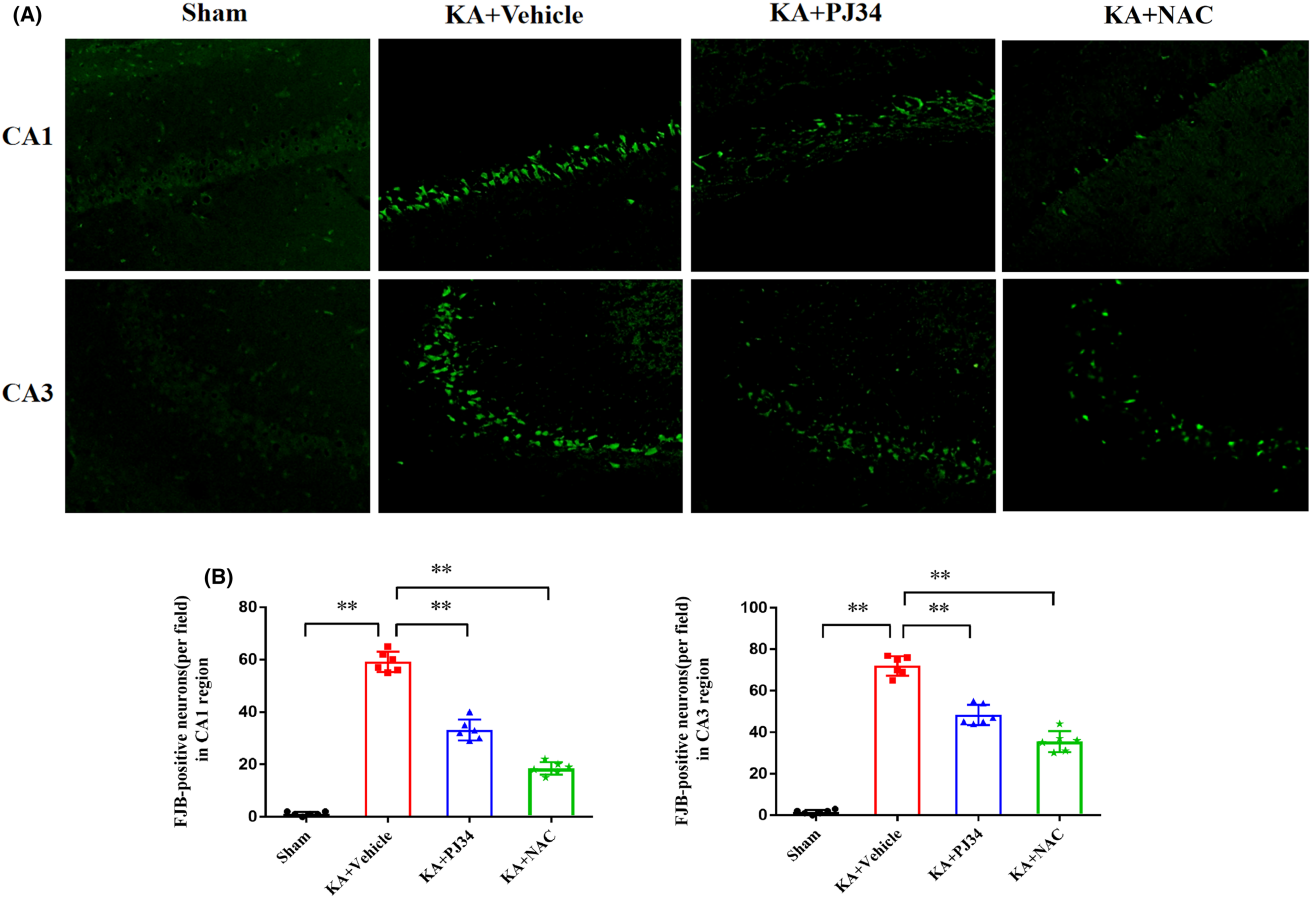
Administration of Pj34 and NAC alleviates hippocampal neuronal loss in KA‐induced epileptic rats. (A) Representative hippocampal sections stained with FJB show degenerating neurons from different groups. (B) Quantitative analysis reveals significant numbers of FJB‐positive neurons presented in CA1 and CA3 regions after status epilepticus (SE) induction, and treatment with PJ34 and NAC reduces FJB‐positive neurons. Scale bar, 50 μm (*n* = 6 per group, **p < 0.01). KA, kainic acid; NAC, N‐acetylcysteine.

FJB staining was used to evaluate the effects of PJ34 and NAC on neuronal loss in KA‐induced epileptic rats (*n* = 6 per group; Figure 7). Hippocampal neurons in the CA1 and CA3 regions were significantly damaged in the KA+ vehicle group when compared to those in the sham group (CA1, *p* < 0.01; CA3, *p* < 0.01). Administration of PJ34 (CA1, *p* < 0.01; CA3, *p* < 0.01) and NAC (CA1, *p* < 0.01; CA3, *p* < 0.01) significantly rescued KA‐induced hippocampal neuronal damage.

### 3.7 PJ34 and NAC modulated expression levels of parthanatos‐related proteins in epileptic rats

**FIGURE 8:**
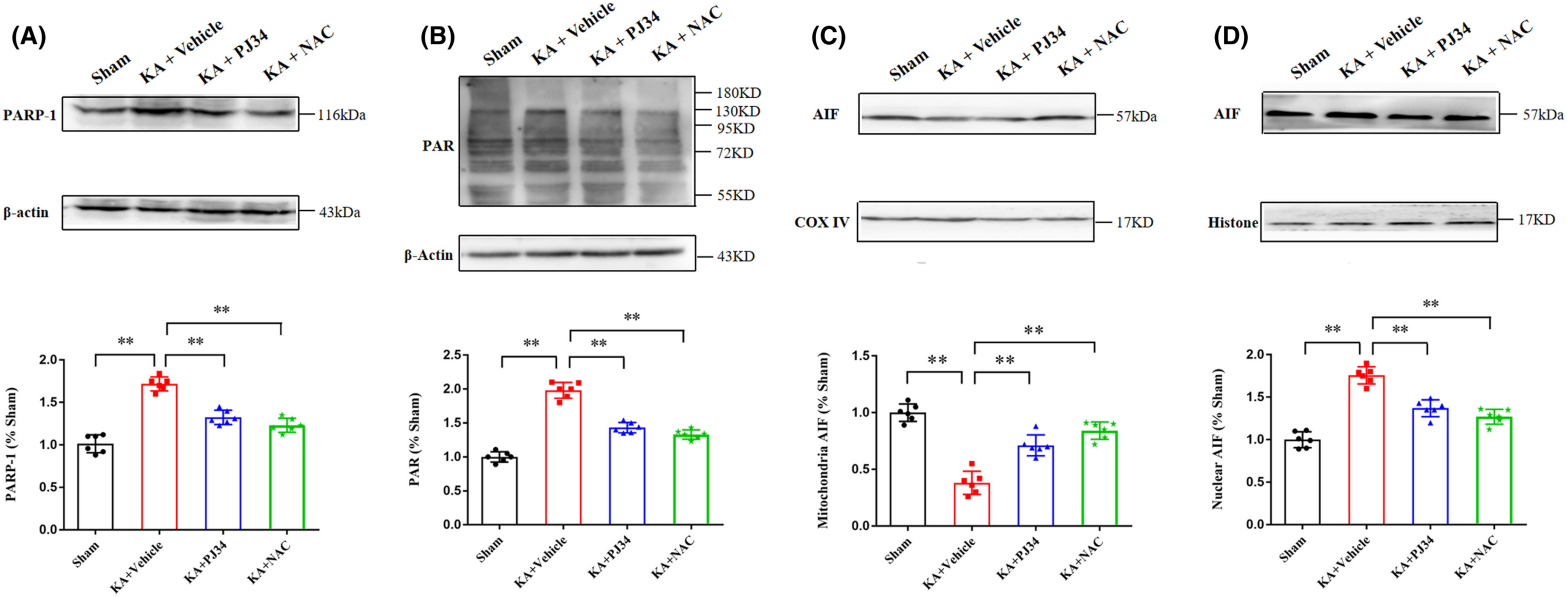
Administration of PJ34 and NAC modulates parthanatos‐related protein expression in KA‐induced epileptic rats. (A, B) Administration with PJ34 and NAC significantly decreases the increased PARP‐1 and PAR polymer expression in status epilepticus (SE)‐induced rats. (C, D) The expression level of AIF decreases in mitochondrial but increases in the nucleus of SE‐induced rats, which can be reversed by PJ34 and NAC administration (*n* = 6 per group, **p < 0.01). KA, kainic acid; NAC, N‐acetylcysteine.

On Day 3 post‐SE induction, Western blot analysis was performed to evaluate the effects of PJ34 and NAC on parthanatos‐related protein expression (*n* = 6 per group; Figure 8). The hippocampus of KA‐induced epileptic rats exhibited increased expression of PARP‐1 and PAR polymer, along with elevated AIF translocation from the mitochondria to the nucleus; administration of PJ34 and NAC significantly alleviated these changes. The results of the in vivo experiment indicated that parthanatos might participate in hippocampal neuronal damage following SE induction, and the PARP‐1 inhibitor PJ34 and antioxidant NAC exhibited neuroprotective effects potentially mediated via parthanatos inhibition.

## 4 DISCUSSION

The treatment of epilepsy has mainly relied on drug therapy to control epileptic symptoms. Although various antiepileptic drugs (AEDs) are available, 30%–40% of patients exhibit drug resistance, presenting uncontrollable epileptic seizures, thereby resulting in refractory epilepsy.^22^ Corresponding molecular pathological changes have been established in the clinical diagnosis of epilepsy. At this stage, treatment with AEDs has failed to effectively control seizure activity. Therefore, exploring the pathogenesis of epilepsy is critical, potentially affording novel approaches for epileptogenic inhibition.

Elucidating the mechanism of seizure‐induced cell death and examining potential interventional targets will provide new directions for inhibiting the occurrence and development of epilepsy. Glutamate is a primary mediator of excessive excitatory neurotransmission induced by neuronal excitotoxicity following epileptic seizures.^17^ We performed an in vitro study using glutamate to induce HT22 cell damage for simulating hippocampal neuronal death following epilepsy and determined whether parthanatos participates in glutamate‐induced neuronal death. Herein, we examined the role of PARP‐1 using the PARP‐1 inhibitor PJ34 and siRNA‐mediated PARP‐1 knockdown on glutamate‐induced HT22 cell damage and parthanatos‐related events. Our results revealed that glutamate‐induced HT22 cell death was accompanied by increased PARP‐1 expression, excessive PAR polymer accumulation, nuclear translocation of AIF, and a decline in the percentage of mitochondrial membrane potentials. Inhibition of PARP‐1 effectively protected HT22 cells against glutamate‐mediated toxic effects, attenuated levels of PARP‐1 and PAR polymers, and alleviated AIF translocation into the nucleus and mitochondrial depolarization. In vivo experiments using KA‐induced epileptic rats showed that PARP‐1 inhibition could effectively protect hippocampal neurons from damage and inhibit parthanatos‐related events. Collectively, these findings support that parthanatos might participate in glutamate‐induced HT22 cell injury and seizure‐induced hippocampal neuronal death.

Parthanatos was found to be involved in multiple diseases, including cerebral hypoxia/ischemia, inflammation, diabetes, and trauma.^16^, ^23^, ^24^, ^25^ PARP‐1 overactivation is considered the initiating step in parthanatos. PARP‐1 is regarded as a “DNA damage sensor.” Under normal physiological conditions, PARP‐1 is activated by DNA strand breakage to repair cellular DNA damage and maintain cellular homeostasis.^26^ Under pathological conditions, overactivation of PARP‐1 following serious DNA damage results in excessive synthesis and accumulation of PAR polymer, which translocates into the cytoplasm to induce cytotoxic effects, resulting in parthanatos.^27^

PAR polymers are toxic to cells, and the toxicity of PAR is related to the length and complexity of the PAR polymer.^10^, ^28^ PAR polymers with ˃ 60 ADP‐ribose units are more toxic than less complex polymers.^10^ PAR is a negatively charged polymer that can alter the internal biochemical characteristics of the receptor and regulate its structure and function.^29^ PAR is synthesized in the nucleus and subsequently transferred to mitochondria, resulting in phosphatidylserine turnover to the ectoplasmic membrane, which induces mitochondrial depolarization and mitochondrial permeability transition pore opening, which leads to untruncated AIF release from the mitochondria.^10^, ^30^ AIF is a widely expressed mitochondrial protein that translocates from the mitochondria to the nucleus to induce DNA fragmentation.^31^ To date, the mechanism through which AIF induces DNA fragmentation remains unclear. A recent study has identified a PARP‐1‐dependent AIF‐associated nuclease, macrophage migration inhibitory factor, that cleaves genomic DNA into large fragments.^32^

PARP‐1 is directly activated by chromosomal DNA strand breaks.^27^ ROS are important mediators of DNA damage via the oxidation of nucleoside bases (such as 8‐oxguanine formation).^33^ Ma et al have found that deoxypodophyllotoxin (DPT) triggers excessive PARP‐1 expression and synthesis of PAR polymer in glioma cells. The authors also confirmed that DPT‐induced ROS production contributed to the PARP‐1 activation.^20^ In epilepsy, excessive ROS production can be induced during epileptic discharge. Increased ROS production was initially confirmed in brain homogenates of an in vivo epileptic model.^34^ This finding was supported by fluorescence staining in vitro.^35^, ^36^ Kumari et al have reported that ROS levels were increased in a glutamate‐induced HT22 cell injury model, and the glutamate‐induced oxidative damage was concentration‐dependent.^37^ Our study revealed that glutamate could induce TH22 cell death in a time‐dependent manner, accompanied by intracellular ROS production, 8‐OHdG generation, and decreased GSH levels. Pretreatment with NAC rescued glutamate‐induced HT22 cell injury, decreased the intracellular ROS production and 8‐OHdG generation, increased the GSH level, and inhibited glutamate‐induced parthanatos‐related protein events. The neuroprotective effect of NAC mediated via parthanatos inhibition was further confirmed in an animal model of epilepsy. Therefore, these results indicate that ROS may be the initiating factor of parthanatos. NAC, an antioxidant, may play a neuroprotective role by increasing intracellular GSH levels.

## 5 CONCLUSION

The present study demonstrated that parthanatos participated in glutamate‐induced HT22 cell injury and hippocampal neuronal damage in rats following epileptic seizures. In addition, ROS might be the initiating factor in the process of parthanatos. Our findings highlight the robust neuroprotective potential of pharmacological interventions targeting PARP activity in epilepsy and provide multiple targets that need to be verified in future studies.

## AUTHOR CONTRIBUTIONS

XW performed the cell experiment part and wrote the manuscript. WQ performed the animal experiment part. MM contributed to the analysis of this study. PF and HM contributed to the design of this study. All authors read and approved the final manuscript.

## Funding information

This study was supported in part by the National Natural Science Foundation of China (81871008) and National Key R&D Program of China (2017YFC0110304).

## CONFLICT OF INTEREST

The authors state no conflict of interest.

## Data Availability

The data that support the findings of this study are available from the corresponding author upon reasonable request.
